# Food Insecurity and Associated Factors in Brazilian Undergraduates during the COVID-19 Pandemic

**DOI:** 10.3390/nu14020358

**Published:** 2022-01-14

**Authors:** Bruna Leal Lima Maciel, Clélia de Oliveira Lyra, Jéssica Raissa Carlos Gomes, Priscilla Moura Rolim, Bartira Mendes Gorgulho, Patrícia Simone Nogueira, Paulo Rogério Melo Rodrigues, Tiago Feitosa da Silva, Fernanda Andrade Martins, Tatiane Dalamaria, Thanise Sabrina Souza Santos, Doroteia Aparecida Höfelmann, Sandra Patricia Crispim, Betzabeth Slater, Alanderson Alves Ramalho, Dirce Maria Marchioni

**Affiliations:** 1 Graduate Program in Nutrition, Department of Nutrition, Federal University of Rio Grande do Norte, Natal 59078-970, RN, Brazil; clelialyra@gmail.com (C.d.O.L.); jessicacarlosnutri@gmail.com (J.R.C.G.); priscilla.rolim@ufrn.br (P.M.R.); 2 Department of Food and Nutrition, Nutrition Faculty, Federal University of Mato Grosso, Cuiabá 78060-900, MT, Brazil; bartira.gorgulho@gmail.com (B.M.G.); patricianogueira.ppj@gmail.com (P.S.N.); prmr84@gmail.com (P.R.M.R.); 3 Graduate Program in Public Health, Federal University of Acre, Rio Branco 69920-900, AC, Brazil; tiago.feitosa@sou.ufac.br (T.F.d.S.); alanderson.ramalho@ufac.br (A.A.R.); 4 Health and Sports Science Center, Nutrition Course, Federal University of Acre, Rio Branco 69920-900, AC, Brazil; fernanda.martins@ufac.br; 5 Graduate Program in Nutrition and Public Health, Faculty of Public Health, University of São Paulo, São Paulo 05508-000, SP, Brazil; tatianedalamaria@usp.br; 6 Nutrition Interventions Research Group, School of Nursing, Federal University of Minas Gerais, Belo Horizonte 30130-100, MG, Brazil; thanisesouza@gmail.com; 7 Center for Epidemiological Research in Nutrition and Health (NUPENS), Faculty of Public Health, University of São Paulo, São Paulo 01246-904, SP, Brazil; 8 Department of Nutrition, Federal University of Paraná, Curitiba 80210-170, PR, Brazil; doroteia.hofelmann@ufpr.br (D.A.H.); crispim@ufpr.br (S.P.C.); 9 Nutrition Department, School of Public Health, University of São Paulo, São Paulo 01246-904, SP, Brazil; bslater@usp.br (B.S.); marchioni@usp.br (D.M.M.)

**Keywords:** diet quality, nutrition, food security

## Abstract

Undergraduates may face challenges to assure food security, related to economic and mental distress, especially during the COVID-19 pandemic. This study aimed to assess food insecurity and its associated factors in undergraduates during the COVID-19 pandemic. An online cross-sectional study was conducted from August 2020 to February 2021 with 4775 undergraduates from all Brazilian regions. The questionnaire contained socio-economic variables, the validated Brazilian food insecurity scale, and the ESQUADA scale to assess diet quality. The median age of the students was 22.0 years, and 48.0% reported income decreasing with the pandemic. Food insecurity was present in 38.6% of the students, 4.5% with severe food insecurity and 7.7% moderate. Logistic regressions showed students with brown and black skin color/race presented the highest OR for food insecurity; both income and weight increase or reduction during the pandemic was also associated with a higher OR for food insecurity, and better diet quality was associated with decreased OR for food insecurity. Our study showed a considerable presence of food insecurity in undergraduates. Policy for this population must be directed to the most vulnerable: those with brown and black skin color/race, who changed income during the pandemic, and those presented with difficulties maintaining weight and with poor diet quality.

## 1. Introduction

Food insecurity exists when there is deprivation or uncertainty about access to quality food in enough quantity to live an active and healthy life [1]. It is associated with several chronic diseases, poor mental health, and poor diet quality [2,3,4,5] and remains an important public and social health problem in countries with different levels of economic development, which were accentuated by the effects COVID-19 pandemic [6].

According to the Food and Agriculture Organization of the United Nations (FAO), in a recent global report of food insecurity and nutrition, in 2020, there was an increase in food insecurity to 30.4%, representing the total food insecurity of the previous five years combined. Thus, almost one in three people in the world did not have access to adequate food in 2020—an increase of 320 million people in just one year, from 2.05 to 2.37 billion. Nearly a tenth of the world’s population—up to 811 million people—faced hunger in 2020. The increase in moderate or severe food insecurity was more evident in Latin America and the Caribbean, followed by Africa and Asia. Even in North America and Europe, where the lowest rates of food insecurity are found, the prevalence of FI has increased for the first time [6].

The coronavirus pandemic spread rapidly and intensely and found fertile ground for its dissemination and community transmission in Brazil [7]. According to World Health Organization (WHO) statistics, the country is the third with the highest number of infected people and the second with the highest number of deaths [8]. This pandemic scenario has amplified existing social, economic, racial, and gender inequities, further compromising the guarantee of the human right to adequate food and the achievement of food and nutrition security, especially among the most vulnerable [7,9,10].

In Brazil, strategies to prevent coronavirus transmission and the exponential increase in the number of infected people emphasized social isolation, implementation of remote work, and suspension of in-person classes in schools and universities throughout the country [11,12].

Studies consistently showed that university students have higher rates of food insecurity and may be disproportionately impacted by the COVID-19 pandemic [13,14]. In addition, food insecurity in university students is associated with poorer food quality, poor health, increased risk of chronic diseases, poor mental health, and lower academic performance [15,16,17,18,19,20,21,22,23]. Food insecurity is even more worrisome for a population where stress is highly prevalent because food-insecure university students may face additional challenges that can exacerbate mental distress, such as worrying about grade point averages and balancing school with work and extracurricular activities [21].

Although it has been well-established that university students may face food insecurity, and the COVID-19 pandemic is building on this evidence, literature is limited on the food-security changes that university students are experiencing during the COVID-19 pandemic. When universities transitioned to remote learning without in-person classes, many students experienced changes in their lives and financial conditions. Some students may have been in situations where food insecurity has aggravated, while others may have improved. Given the potential and long-term nature of the COVID-19 pandemic, it is necessary to fully understand food insecurity and its associated factors in undergraduate students at Brazilian universities. Thus, the present study aimed to assess food insecurity and its associated factors in undergraduates from Brazilian universities during the COVID-19 pandemic. Our study showed a considerable presence of food insecurity, especially in those of brown and black skin color/race, who changed income during the pandemic, and those who faced difficulties maintaining weight and poor diet quality.

## 2. Materials and Methods

### 2.1. Ethics

This study was carried out at universities in 5 different states, one from each Brazilian region: Acre, Rio Grande do Norte, São Paulo, Mato Grosso, and Paraná. Each site had approved the study protocol at local institutional ethical review boards at the Federal University of Acre (CAAE 36814320.9.0000.5010, #4.267.655), Federal University of Rio Grande do Norte (CAAE: 35918620.7.0000.5292, #4.391.606), University of São Paulo (CAAE 36402820.9.0000.5421, #4.232.859), Federal University of Mato Grosso (CAAE 36582820.0.0000.8124, #4.242.364), and the Federal University of Paraná (CAAE 36250320.2.0000.0102, #4.256.436). All participants registered online consent to participate in the study.

### 2.2. Study Design and Participants

**Figure 1:**
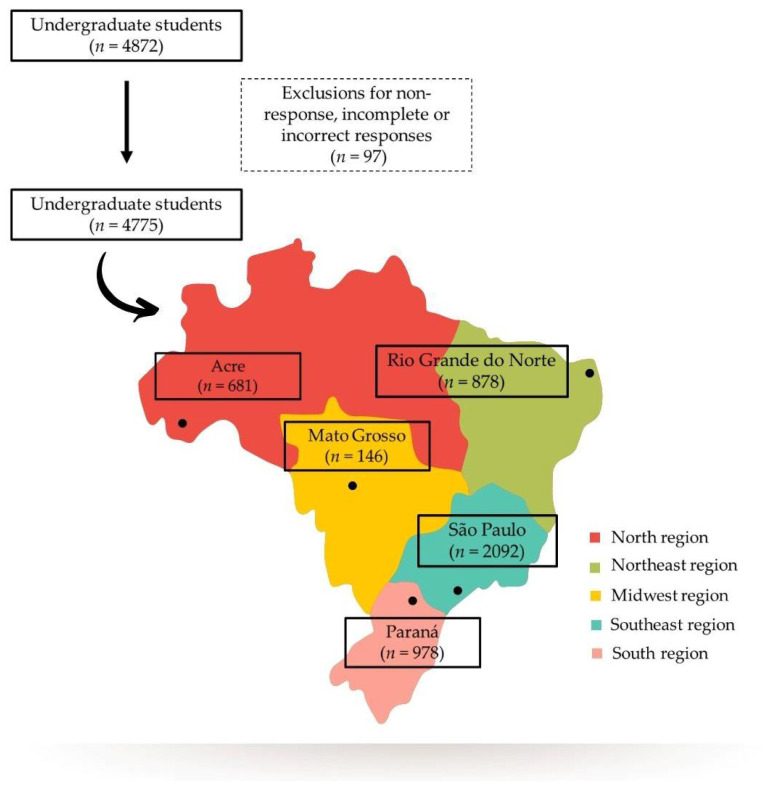
Flowchart of studied undergraduates (*n* = 4775), considering the study sites.

This is a cross-sectional study, with data collection conducted from August 2020 to February 2021, corresponding to the middle of first the COVID-19 wave and beginning of the second wave in Brazil [8]. The study population consisted of undergraduate students from the following universities, located in one of the 5 different Brazilian regions: the Federal University of Acre (north region), Federal University of Rio Grande do Norte (northeast region), University of São Paulo (southeast region), Federal University of Mato Grosso (midwest region), and Federal University of Paraná (south region). Students regularly enrolled in an undergraduate course were eligible for the study. Those who consented to participate in the study and answered the online questionnaire were included (*n* = 4872). A total of 97 students were excluded from the study due to non-response or incomplete responses concerning the food insecurity scale used in the study, totaling 4775 participants (Figure 1).

The online questionnaire was prepared using the Google Forms platform and sent to the students’ institutional electronic addresses (e-mails). The aims of the research were also publicized using institutional social media to promote participation. The questionnaire was a compilation of socio-economic variables (including race, family income, and income change during the pandemic) and validated scales.

### 2.3. Food Insecurity

Food insecurity was assessed using the adapted and validated Brazilian food-insecurity scale [24,25]. The scale is based on the sum of affirmative answers to 14 questions. The scores fall within cutoff points equivalent to the graded theoretical constructs about food security: food security (total score = 0), mild food insecurity (in households with people <18 years old, total scores 1–5; households without people <18 years old, total scores 1–3), moderate food insecurity (in households with people <18 years old, total scores 6–9; households without people <18 years old, total scores 4–5), and severe food insecurity (in households with people <18 years old, total scores 10–14; households without people <18 years old, total scores 6–8).

The scale has excellent cost effectiveness and has been used in several studies [15,26,27,28,29]. Its application and analysis have shown that there are common aspects to the different socio-cultural contexts of food insecurity represented in the scale, including (1) psychological component—anxiety or doubt about future availability of food in the house to meet the needs of residents; (2) food quality—impairment of socially established preferences about food and its variety at home; (3) quantitative reduction of food among adults; (4) quantitative reduction of food among children; and (5) hunger—when someone goes without food all day due to lack of money to buy food [30,31,32].

### 2.4. Self-Referred Changes in Weight and Diet Quality Assessment

Self-referred changes in weight during the pandemic were registered in the online form, and the validated diet quality scale (ESQUADA) was applied to assess diet quality. The scale presents 15 items, including eating practices (such as replacing meals with snacks and cooking habits) and fresh, minimally processed, and ultra-processed food consumption. Items present alternative answers covering frequency of food consumption, meal cooking, and meal replacement. From the answers, diet quality scores were calculated using the item response theory. These scores were categorized into five levels of diet quality as follows: “very poor” (scores ≤ 150), “poor” (scores > 150 and ≤200), “good” (scores > 200 and ≤275), “very good” (scores > 275 and ≤375), and “excellent” (scores > 375) [33].

### 2.5. Statistical Analysis

Categorical variables were presented as absolute and relative frequencies, and the chi-square test was used to evaluate the frequency distributions of categorical variables. Continuous variables were tested for normality using the Kolmogorov–Smirnov test. Data without normal distribution were presented as median (Q1–Q3) and analyzed using the Kruskal–Wallis test.

Variables that showed a significant association in the univariate analysis with food insecurity were used for logistic regression models, primarily in bivariate analysis, exploring the effect of a single variable on food insecurity, with the unadjusted odds ratios (OR) and their respective 95% confidence intervals (95% CI) demonstrated. Then, logistic regression models were calculated, considering food insecurity as a dependent variable. The adjustment of the final model was guaranteed by observing the Omnibus test, with *p*-values less than 0.01, and the Hosmer and Lemeshow test, considering *p*-values greater than 0.01. Race, income change during the pandemic, weight change during the pandemic, and the ESQUADA classification were included in the final model as independent variables. Sex, age, and study site were used in the final model as adjustment variables. The adjusted odds ratios (AOR) and their respective 95% CI were presented. Given the large sample size, the significance level was set at 1% to avoid type 1 errors. Data analysis was performed using the Statistical Package for Social Sciences version 11.5 (SPSS Inc., Chicago, IL, USA).

## 3. Results

### 3.1. Characterization of the Studied Undergraduates

The characterization of the undergraduate students is shown in Table 1. The undergraduates’ median age was 22.0 (20.0–26.0) years and varied slightly among the studied sites (chi-square, *p* < 0.001). Most respondents in the studied universities were women (66.6%) and declared white (61.0%). Family income was mainly around 1–6 minimum wages, and 48.0% of the students reported less income with the pandemic (chi-square, *p* < 0.001). In addition, 55.6% of the students increased their weight during the pandemic (Table 1, chi-square, *p* < 0.001). The ESQUADA showed that 8.5% of the students presented poor diet quality, 52.0% good, and 37.1% very good diet quality (Table 1, chi-square, *p* < 0.001).

### 3.2. Food Security

**Figure 2:**
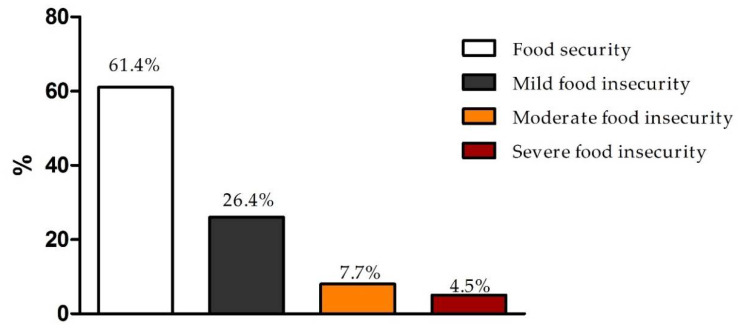
Food insecurity in undergraduates (*n* = 4775), assessed using the Brazilian Food Insecurity Scale. Chi-square test, *p* < 0.001.

Considering food security, 26.4% of the students presented mild food insecurity, 7.7% moderate food insecurity, and 4.5% severe food insecurity (Figure 2). The bivariate association analysis (Table 2) showed that most of the students presenting any food insecurity declared brown or black skin color/race and reduced family income during the pandemic (chi-square test, *p* < 0.001). Students with severe food insecurity presented more weight reduction during the pandemic (38.2%) when compared to those with food security (27.3%), mild food insecurity (25.9%), and moderate food insecurity (27.8%) (chi-square test, *p* < 0.001; Table 2). Diet quality was also associated with food security; while 12.8% of the students with severe food insecurity presented poor diet quality, 8% of those with food security showed poor diet quality (chi-square test, *p* < 0.001; Table 2).

The logistic regressions (Table 3) further explored the observed associations: students declared with brown skin color/race presented an AOR = 1.93 (95% CI = 1.67–2.24) for food insecurity, and those declared with black skin color/race presented an AOR = 2.89 (95% CI = 2.27–3.68). Income increase or reduction during the pandemic also increased the odds of food insecurity (AOR = 1.83, 95% CI = 1.47–2.28; AOR = 2.78, 95% CI = 2.43–3.18, respectively). Weight change during the pandemic was also associated with food insecurity. Students who lost weight presented an AOR =1.44 (95% CI = 1.16–1.79) for food insecurity and those who gained weight an AOR = 1.36 (95% CI = 1.11–1.67). Better diet quality decreased the odds of food insecurity: students with very good and excellent diet quality presented an AOR = 0.46, 95% CI = 0.27–0.79; AOR = 0.26, 95% CI = 0.11–0.65, respectively.

## 4. Discussion

This study aimed to assess food insecurity and its associated factors in undergraduate students from Brazilian universities during the COVID-19 pandemic. Our data showed that food insecurity was present in a large number the students, and this was associated with skin color/race, changes in income during the pandemic, and nutritional variables, such as weight change and diet quality. The harmful impacts of the COVID-19 pandemic on food security and nutrition were expected to occur. These impacts were higher in those most vulnerable populations with less access to healthy foods and more social inequities, which are some of the risk factors in Brazil [28].

Before the COVID-19 pandemic, in 2017–2018, food insecurity was present in 36.6% of Brazilian households [34]. Data collected in December 2020 indicated food insecurity was present in 55.2% of Brazilian households [29], corroborating the expected increase in food insecurity due to the COVID-19 pandemic. Data from our study indicated that undergraduates also represent a vulnerable population to food insecurity, with 38.6% experiencing any kind of food insecurity. This value could be even higher if only those students living in campus were studied, as was the case of the study from Araujo et al. [15], where food insecurity was present in 84.5% of the students.

The higher odds of food insecurity in brown and black skin color/race undergraduates were consistent with the social vulnerability of that population. In the last 15 years, affirmative policies increased the number of black skin color/race undergraduates in public universities in Brazil. Nevertheless, it is essential to reinforce policies to ensure their permanence in the University, and thus, food security must be guaranteed. Few studies have addressed food insecurity in Brazilian university students. In 2018, around 70% of students enrolled in federal universities/institutes in Brazil (65 universities and 2 federal institutes) presented a per capita family income of up to 1.5 minimum wages [35]. Indeed, our results reinforce that studying this most vulnerable population is essential, showing that students declared with brown and black skin color/race presented the highest odds for food insecurity.

The social and economic impacts of the pandemic might have increased food insecurity in undergraduate students. Our study showed that income change to less or more than before the pandemic were both associated with a higher chance for food insecurity. These results might be explained by the fact that less income represents a threat to food security, as addressed in other studies [26,27]. The income change to more than before the pandemic association with food insecurity might be explained by the fact that some of the students’ families might have received the government’s emergency aid (around $111) designated for those with lower income and without formal employment [11]. Data from the Brazilian population during the COVID-19 pandemic indicate that those who received the emergency aid where the most affected by food insecurity [29], and 63% of those who received the emergency aid used it to buy food [36]. This is probably the case of our studied population, and low-income students who already face social adversities, in addition to the challenges to education, might be even more vulnerable to the effects of the pandemic, the political crisis, and economic recession in Brazil. Furthermore, during the pandemic, food inflation in Brazil has risen significantly, and the emergency aid might not have been enough to guarantee food security to those most vulnerable [37].

Furthermore, institutional university restaurants, which supplied students with healthy/adequate/secure meals at low prices and was free to the most vulnerable students, were closed as part of the measures for social isolation during the pandemic. Other forms to guarantee the access to food security and nutrition, such as farmers’ markets, were also closed in the pandemic. Thus, these measures might have impacted the quality of the students’ diet. Indeed, our results have shown that better overall diet quality was associated with lower chances for food insecurity. The SQUADA scale used in the present study assesses not only foods/nutrients but specially feeding practices, such as preparing foods, substituting meals prepared at home for fast foods, and ultra-processed foods [33]. The results from our study are unprecedented, as they demonstrate the association of food security to better diet quality, measured in wider context, considering food preparation and consumption. Furthermore, improving the quality of diets using culinary skills to promote cooking self-efficacy and food agency might be a way of reducing food insecurity and perceived stress in university students, as successfully addressed by Matias et al. [38]. Thus, programs addressing cooking self-efficacy and food agency might be helpful to our studied population.

Students who lost or gained weight during the pandemic also presented more food insecurity. This fact is possibly also associated to the quality of diets consumed. Studies have consistently demonstrated that consuming a diet based in fast foods or ultra-processed foods is associated with overweight and chronic diseases [39,40]. In our study, consuming a diet with less of these foods was associated with less chance for food insecurity. In addition, students who lost weight during the pandemic might have also experienced more perceived stress and food deprivation, which explains the association with food insecurity.

The limitations of our study must be mentioned. The online data collection could have restricted the participation of those who did not have internet access. Nevertheless, by the time data collection finished (February 2021), most public universities had given support, such as scholarships for internet or notebook acquisition, to those most vulnerable students to guarantee online class attendance. The non-probabilistic sampling might have given selection bias because the motivation to respond to the online form could have been higher in those most affected by the pandemic. However, the identification and comprehension of these individuals were part of the research. An additional limitation of the study is the timing of the survey (fall vs. spring semester). For U.S. students, the prevalence of food insecurity was higher during the second semester of the academic year [41]. However, our study did not address this because the pandemic changed the semesters’ continuity in each studied university differently.

The best practices for web surveys were followed to ensure reliability [42]. The strengths of the study are the use of validated scales to measure food insecurity [25] and diet quality [33], data coming from universities from all the Brazilian regions, and elucidating a population rarely studied for food insecurity in the context of the COVID-19 pandemic. In addition, the results may contribute to policy planning for this group of students, who might be experiencing social and economic difficulties due to the pandemic that can negatively impact education.

## 5. Conclusions

Our data showed a considerable presence of food insecurity during the COVID-19 pandemic in undergraduates. Food insecurity was associated with brown and black skin color/race, those who changed income during the pandemic, and those presented with difficulties maintaining weight and with poor diet quality.

Food security and nutrition, beyond guaranteeing life quality and dignity, are related to better health, protecting against COVID-19 and other diseases [9,43]. Thus, food insecurity must be tackled, especially in the young generations. In Brazilian undergraduates, our study indicated that policy must be directed to the most vulnerable: those declared with brown or black skin color/race, who have changed income during the pandemic, and those presented with difficulties in maintaining weight and with poor diet quality. Further studies should address programs to promote food security, nutritional education, and access to healthy food in the studied population. Policies should also address university restaurants in keeping and strengthening their services during the pandemic to guarantee access to quality/safe food to undergraduates.

## Figures and Tables

**Table 1:** Characterization of the undergraduates (*n* = 4775), according to study site.

Variables	Total	Acre	Mato Grosso	Paraná	Rio Grande do Norte	São Paulo	*p*-Value
Age, median (Q1–Q3)	22.0 (20.0–26.0)	22.0 (20.0–25.0)	25.0 (21.8–36.3)	22.0 (20.0–26.0)	24.0 (21.0–29.3)	22.0 (20.0–25.0)	<0.001 ^1^
Sex, *n* (%)							
Male	1596 (33.4)	219 (32.2)	51 (34.9)	313 (32.0)	307 (35.0)	706 (33.7)	0.634 ^2^
Female	3179 (66.6)	462 (67.8)	95 (65.1)	665 (68.0)	571 (65.0)	1386 (66.3)	
Total	4775 (100.0)	681 (100.0)	146 (100.0)	978 (100.0)	878 (100.0)	2092 (100.0)	
Race, *n* (%)							
Asiatic	165 (3.5)	10 (1.5)	2 (1.4)	30 (3.1)	4 (0.5)	119 (5.7)	<0.001 ^2^
White	2914 (61.0)	158 (23.2)	82 (56.2)	761 (77.8)	444 (50.6)	1469 (70.2)	
Indigenous	15 (0.3)	7 (1.0)	0 (0.0)	0 (0.0)	2 (0.2)	6 (0.3)	
Brown	1326 (27.8)	416 (61.1)	52 (35.6)	142 (14.5)	346 (39.4)	370 (17.7)	
Black	333 (7.0)	77 (11.3)	10 (6.8)	41 (4.2)	82 (9.3)	123 (5.9)	
NI/NWI	22 (0.5)	13 (1.9)	0 (0.0)	4 (0.4)	0 (0.0)	5 (0.2)	
Total	4775 (100.0)	681 (100.0)	146 (100.0)	978 (100.0)	878 (100.0)	2092 (100.0)	
Family income in minimum wages, *n* (%) ^3^							
None	130 (2.7)	30 (4.4)	5 (3.4)	8 (0.8)	47 (5.4)	40 (1.9)	<0.001 ^2^
0–1	704 (14.7)	242 (35.5)	24 (16.4)	92 (9.4)	198 (22.6)	148 (7.1)	
1–3	1471 (30.8)	209 (30.7)	33 (22.6)	329 (33.6)	326 (37.1)	574 (27.4)	
3–6	1034 (21.7)	82 (12.0)	22 (15.1)	259 (26.5)	177 (20.2)	494 (23.6)	
6–9	524 (11.0)	41 (6.0)	20 (13.7)	114 (11.7)	57 (6.5)	292 (14.0)	
9–12	330 (6.9)	16 (2.3)	20 (13.7)	76 (7.8)	28 (3.2)	190 (9.1)	
12–15	220 (4.6)	11 (1.6)	11 (7.5)	48 (4.9)	19 (2.2)	131 (6.3)	
>15	323 (6.8)	11 (1.6)	11 (7.5)	52 (5.3)	26 (3.0)	223 (10.7)	
NI/NWI	39 (0.8)	39 (5.7)	0 (0.0)	0 (0.0)	0 (0.0)	0 (0.0)	
Total	4775 (100.0)	681 (100.0)	146 (100.0)	978 (100.0)	878 (100.0)	2092 (100.0)	
Income change during the pandemic, *n* (%)							
No	1975 (41.4)	296 (43.5)	79 (54.1)	398 (40.7)	329 (37.5)	873 (41.7)	<0.001 ^2^
Yes, for more	464 (9.7)	81 (11.9)	10 (6.8)	98 (10.0)	103 (11.7)	172 (8.2)	
Yes, for less	2291 (48.0)	265 (38.9)	51 (34.9)	482 (49.3)	446 (50.8)	1047 (50.0)	
NI/NWI	45 (0.9)	39 (5.7)	6 (4.1)	0 (0.0)	0 (0.0)	0 (0.0)	
Total	4775 (100.0)	681 (100.0)	146 (100.0)	978 (100.0)	878 (100.0)	2092 (100.0)	
Weight change during the pandemic, *n* (%)							
No	583 (12.6)	78 (11.9)	27 (19.4)	129 (13.6)	71 (8.4)	278 (13.6)	<0.001 ^2^
Yes, for less	1273 (27.4)	182 (27.7)	34 (24.5)	249 (26.3)	229 (27.1)	579 (28.2)	
Yes, for more	2578 (55.6)	384 (58.4)	74 (53.2)	511 (54.0)	524 (62.0)	1085 (52.9)	
NI/NWI	204 (4.4)	14 (2.1)	4 (2.9)	57 (6.0)	21 (2.5)	108 (5.3)	
Total	4638 (100.0)	658 (100.0)	139 (100.0)	946 (100.)	845 (100.0)	2050 (100.0)	
ESQUADA classification, *n* (%)							
Very poor	64 (1.4)	13 (1.9)	1 (0.7)	13 (1.3)	10 (1.1)	27 (1.3)	<0.001 ^2^
Poor	402 (8.5)	50 (7.4)	8 (5.5)	80 (8.2)	88 (10.1)	176 (8.5)	
Good	2463 (52.0)	391 (58.1)	66 (45.5)	535 (55.2)	472 (54.3)	999 (48.2)	
Very good	1755 (37.1)	217 (32.2)	69 (47.6)	337 (34.7)	295 (33.9)	837 (40.4)	
Excellent	48 (1.0)	2 (0.3)	1 (0.7)	5 (0.5)	5 (0.6)	35 (1.7)	
Total	4732 (100.0)	673 (100.0)	145 (100.0)	970 (100.0)	870 (100.0)	2074 (100.0)	

^1^*p*-value for the Kruskal Wallis test. ^2^*p*-value for the chi-square. ^3^ The minimum wage in Brazil is R$ 1100, around $212. NI/NWI, not informed/did not wish to inform.

**Table 2:** Food security of undergraduate students (*n* = 4775) according to social, anthropometric, and food variables during the pandemic.

Variables	Total	Food Security	Mild Food Insecurity	Moderate Food Insecurity	Severe Food Insecurity	Chi-Square Test, *p*-Value
Race, *n* (%)						
Asiatic	165 (3.5)	124 (4.2)	32 (2.5)	6 (1.6)	3 (1.4)	<0.001
White	2914 (61.0)	2030 (69.2)	644 (51.1)	151 (41.3)	89 (41.6)	
Indigenous	15 (0.3)	6 (0.2)	5 (0.4)	3 (0.8)	1 (0.5)	
Brown	1326 (27.8)	625 (21.3)	454 (36.0)	154 (42.1)	93 (43.5)	
Black	333 (7.0)	135 (4.6)	121 (9.6)	49 (13.4)	28 (13.1)	
NI/NWI	22 (0.5)	14 (0.5)	5 (0.4)	3 (0.8)	0 (0.0)	
Total	4775 (100.0)	2934 (100.0)	1261 (100.0)	366 (100.0)	214 (100.0)	
Family income change during the pandemic, *n* (%)						
No	1975 (41.4)	1448 (49.4)	388 (30.8)	87 (23.8)	52 (24.3)	<0.001
Yes, for more	464 (9.7)	268 (9.1)	119 (9.4)	49 (13.4)	28 (13.1)	
Yes, for less	2291 (48.0)	1198 (40.8)	738 (58.5)	223 (60.9)	132 (61.7)	
NI/NWI	45 (0.9)	20 (0.7)	16 (1.3)	7 (1.9)	2 (0.9)	
Total	4775 (100.0)	2934 (100.0)	1261 (100.0)	366 (100.0)	214 (100.0)	
Weight change during the pandemic, *n* (%)						
No	583 (12.6)	409 (14.3)	129 (10.6)	26 (7.4)	19 (9.3)	<0.001
Yes, for less	1273 (27.4)	783 (27.3)	315 (25.9)	97 (27.8)	78 (38.2)	
Yes, for more	2578 (55.6)	1550 (54.0)	720 (59.2)	209 (59.9)	99 (48.5)	
NI/NWI	204 (4.4)	127 (4.4)	52 (4.3)	17 (4.9)	8 (3.9)	
Total	4638 (100.0)	2869 (100.0)	1216 (100.0)	349 (100.0)	204 (100.0)	
ESQUADA classification, *n* (%)						
Very poor	64 (1.4)	32 (1.1)	22 (1.8)	5 (1.4)	5 (2.4)	<0.001
Poor	402 (8.5)	232 (8.0)	112 (9.0)	31 (8.5)	27 (12.8)	
Good	2463 (52.0)	1412 (48.5)	717 (57.5)	228 (62.6)	106 (50.2)	
Very good	1755 (37.1)	1195 (41.1)	389 (31.2)	98 (26.9)	73 (34.6)	
Excellent	48 (1.0)	39 (1.3)	7 (0.6)	2 (0.5)	0 (0.0)	
Total	4732 (100.0)	2910 (100.0)	1247 (100.0)	364 (100.0)	211 (100.0)	

NI/NWI, not informed/did not wish to inform.

**Table 3:** Logistic regression for variables associated with food insecurity in Brazilian undergraduates during the COVID-19 pandemic.

	Food Insecurity			
Indepenent Variables	OR (95% CI)	*p*-Value	AOR (95% CI)	*p*-Value
Race				
White	−		−	
Asiatic	0.76 (0.53–1.09)	0.136	0.80 (0.55–1.17)	0.252
Indigenous	3.45 (1.22–9.71)	0.019	2.57 (0.81–8.15)	0.107
Brown	2.58 (2.25–2.94)	<0.001	1.93 (1.67–2.24)	<0.001
Black	3.37 (2.67–4.25)	<0.001	2.89 (2.27–3.68)	<0.001
Income change during the pandemic				
No	−		−	
Yes, for more	2.01 (1.63–2.48)	<0.001	1.83 (1.47–2.28)	<0.001
Yes, for less	2.51 (2.20–2.85)	<0.001	2.78 (2.43–3.18)	<0.001
Weight change during the pandemic				
No	−		−	
Yes, for less	1.47 (1.19–1.82)	<0.001	1.44 (1.16–1.79)	0.001
Yes, for more	1.56 (1.28–1.89)	<0.001	1.36 (1.11–1.67)	0.003
ESQUADA classification				
Very poor	−		−	
Poor	0.73 (0.43–1.24)	0.249	0.73 (0.41–1.29)	0.276
Good	0.74 (0.45–1.22)	0.244	0.72 (0.42–1.23)	0.230
Very good	0.47 (0.28–0.77)	0.003	0.46 (0.27–0.79)	0.005
Excellent	0.23 (0.10–0.55)	0.001	0.26 (0.11–0.65)	0.004

OR, crude odds ratio, from bivariate analysis; AOR, adjusted odds ratio, considering all variables in the model. Sex, age, and study site were included as adjustment variables.

## Data Availability

The data presented in this study are available on request from the corresponding author.
